# Impact of Rapid Viral Testing on Patient Flow and Length of Stay in a Tertiary Pediatric Emergency Department

**DOI:** 10.3390/healthcare14070925

**Published:** 2026-04-02

**Authors:** Tommaso Bellini, Giorgia Iovinella, Martina Virgilio, Marcello Mariani, Roberto Bandettini, Andrea Pastorino, Simona Matarese, Francesca Canzoneri, Carlotta Pepino, Barbara Vanorio, Barbara Tubino, Emanuela Piccotti, Andrea Moscatelli

**Affiliations:** 1 Paediatric Emergency Room and Emergency Medicine Unit, Department of Emergency Medicine, Anesthesia and Critical Care, IRCCS Istituto Giannina Gaslini, 16147 Genoa, Italy; simonamatarese@gaslini.org (S.M.); francescacanzoneri@gaslini.org (F.C.); carlottapepino@gaslini.org (C.P.); barbaratubino@gaslini.org (B.T.); emanuelapiccotti@gaslini.org (E.P.); 2 Department of Neuroscience, Rehabilitation, Ophthalmology, Genetics, Maternal and Child Health (DINOGMI), University of Genoa, 16147 Genoa, Italy; 3672473@studenti.unige.it (G.I.); 6554088@studenti.unige.it (M.V.); 5288131@studenti.unige.it (A.P.); 5749021@studenti.unige.it (B.V.); 3 Infectious Diseases Unit, Department of Pediatrics, IRCCS Istituto Giannina Gaslini, 16146 Genoa, Italy; marcellomariani@gaslini.org; 4 Laboratory of Clinical Analysis, Service Department, IRCCS Istituto Giannina Gaslini, 16146 Genova, Italy; robertobandettini@gaslini.org; 5 Pediatric and Neonatal Intensive Care Unit, Department of Emergency Medicine, Anesthesia and Critical Care, IRCCS Istituto Giannina Gaslini, 16146 Genoa, Italy; andreamoscatelli@gaslini.org

**Keywords:** adenovirus, emergency department overcrowding, fever, influenza, length of stay, pediatric emergency department, point-of-care testing, rapid diagnostic tests, respiratory infections

## Abstract

**Highlights:**

**What are the main findings?**

- Rapid viral testing for influenza and adenovirus was associated with shorter length of stay in a high-volume pediatric emergency department.
- Positive test results were linked to a redistribution of decision-making time, resulting in patients being discharged earlier without an increase in 72 h return visits.

**What are the implications of the main findings?**

- Rapid viral testing may support patient flow optimization and operational efficiency in pediatric emergency departments during epidemic seasons.
- Even modest time savings at the individual level may be meaningful when applied to emergency care systems with high patient throughput.

**Abstract:**

Background. Overcrowding in emergency departments (EDs), particularly pediatric emergency departments (PEDs), remains a significant challenge that affects patient outcomes and the efficiency of healthcare. Rapid diagnostic tests (RDTs) for respiratory viruses could be a promising tool for improving patient management by enabling prompt etiological diagnoses. This study investigated whether positive RDT results for influenza or adenovirus were associated with differences in length of stay (LOS) in a tertiary PED during epidemic seasons. Methods. A retrospective cohort study was conducted at IRCCS Istituto Giannina Gaslini, Genoa, Italy, over two epidemic seasons (December–February, 2023–2025). All consecutive pediatric patients presenting with fever and respiratory symptoms who underwent rapid diagnostic testing for influenza and/or adenovirus during two epidemic seasons were included. LOS was assessed as the time from triage to discharge (TTD) and from physician assignment to discharge (ATD). Patients were stratified by positive versus negative RDT results. Analyses between groups used the Mann–Whitney U-test for continuous variables and chi-square or Fisher’s exact test for categorical variables. A two-tailed *p*-value < 0.05 was considered significant. Results. Of the 1238 patients analyzed, the median age was 3.3 years (IQR 1.4–7.2), with male predominance (58.1%). A total of 330 patients (26.6%) tested positive. Compared with negative results, positive RDTs were associated with shorter median TTD (217.0 vs. 239.0 min, *p* < 0.001) and ATD (66.0 vs. 148.5 min, *p* < 0.001), which was consistent in both the influenza and adenovirus subgroups. No significant difference in 72 h readmission rates was observed between groups. Conclusions. Among children tested with RDTs for influenza and adenovirus, positive results were associated with reduced PED LOS without increasing early return visits. While these findings suggest a potential role in supporting patient flow, conclusions regarding the broader impact on PED overcrowding should be drawn with caution. Further prospective studies, including non-tested controls and additional viral targets, are required.

## 1. Introduction

Overcrowding in both adult and pediatric emergency departments (EDs and PEDs, respectively) is a well-documented and persistent issue that pose a significant challenge to healthcare services [1,2,3]. The demand for emergency services often exceeds the available resources, resulting in long waiting times, reduced patient satisfaction, and potential compromises in the quality of care [1]. Notably, infants and young children have the highest rate of hospital admission due to infectious diseases, followed by patients over the age of 75, particularly during the seasonal respiratory virus season [1,2]. Various strategies have been proposed and implemented to address the misuse of resources, including process improvements, point-of-care tests, policy reforms, and healthcare education [2,3,4,5]. Nevertheless, these measures have often failed to achieve the desired outcomes, leaving PED overcrowding and misuse as critical concerns within healthcare systems globally [2,3,4].

Fever is a common reason for visits to PEDs, accounting for around 20–30% of cases, with admissions increasing during seasonal peaks of viral infections [6,7,8,9,10,11,12]. The majority of respiratory illnesses are viral infections, with influenza, parainfluenza, respiratory syncytial virus, and adenovirus being the most common pathogens. However, symptoms are often non-specific, making clinical diagnosis of the causative agent unreliable and necessitating extensive diagnostic investigations [8,9,12,13]. Consequently, the diagnosis of respiratory infections has relied on molecular methods, specifically multiplex Real-Time Polymerase Chain Reaction (RT-PCR) panel testing [6,11,12]. Although multiplex RT-PCR provides precise diagnoses, it has traditionally been conducted in central laboratories, resulting in prolonged test turnaround times (TAT) and significant impacts on emergency department workflows and care processes [5,7,14]. Consequently, delays in laboratory test results are frequently considered one of the factors contributing to overcrowding in EDs and PEDs, as well as prolonged length of stay (LOS) [6].

In recent years, there has been a growing interest in enhancing and expediting the diagnosis of viral illnesses. The implementation of rapid diagnostic tests (RDTs) has emerged as a promising strategy for improving patient management in both EDs and PEDs [6,7,10,11,12,13,14,15,16,17,18]. Despite the theoretical benefits, the impact of RDTs on operational metrics, such as PED LOS, and their potential to mitigate overcrowding remain subjects of ongoing research. While the impact of influenza RDTs on ED and PED flow has been demonstrated by several studies, the role of adenovirus rapid testing has only been partially investigated [5,6,12,16,17].

Reducing PED LOS may indirectly decrease waiting room congestion, thereby improving patient flow and satisfaction [1,5,6,9,10,11]. Thus, the objective of the present study was to evaluate the association between rapid viral testing for influenza A/B and adenovirus and emergency department operational metrics, including patient flow and LOS. By potentially decreasing LOS, this approach could improve throughput and resource allocation, and offer a partial solution to the challenges of PED overcrowding and misuse.

## 2. Materials and Methods

A retrospective cohort study was conducted at PED of the Istituto di Ricerca e Cura a Carattere Scientifico (IRCCS) Giannina Gaslini. The study analyzed data from pediatric patients who presented to the PED during the last two epidemic seasons (2023–2024, 2024–2025), defined as the period from the first day of December to the last day of February. The IRCCS Giannina Gaslini is a tertiary care children’s hospital serving the Liguria region in north-western Italy. It receives an average of 38,000 PED visits per year from patients aged 0–18 years. All retrospective data were collected from anonymized electronic medical records. The inclusion criteria encompassed all consecutive patients who underwent RDT for adenovirus and/or influenza due to febrile illness accompanied by respiratory symptoms. Patient data were obtained from the RDT device records, and assignment and discharge times were calculated using the PED flow management program. According to the IRCCS Giannina Gaslini’s institutional protocol, RDTs were performed in children presenting with fever and respiratory symptoms, including rhinitis, cough, wheezing, or respiratory distress, regardless of clinical severity. As the decision to perform influenza-only, adenovirus-only, or both RDTs was at the discretion of the treating physician, patient selection may have been influenced by clinical judgment or epidemiological context.

All tests were conducted using the Standard F Antigen Point-of-Care Test Kit produced by Relab, SD BiosensorTM (Corso Perrone, 25r, 16152 Genoa, Italy). The respective sensitivities and specificities for influenza and adenovirus were 97.0% and 97.6%, and 83.3% and 95.5%, respectively [6]. The TAT was approximately 10–15 min, and tests were performed on demand at the bedside, eliminating the need for samples to be sent to a laboratory. The point-of-care test requires no specific expertise and can be performed by healthcare personnel following a brief demonstration.

Data collected included patient demographics (age, sex), test results, LOS in the PED measured in minutes from triage to discharge (TTD) and from the physician assignment to discharge (ATD), and return visits to the PED within 72 h post-discharge. Both TTD and ATD were calculated until PED discharge, which was defined as discharge home or transfer to an inpatient ward. The total hospital stay for admitted patients was not considered.

**Figure 1:**
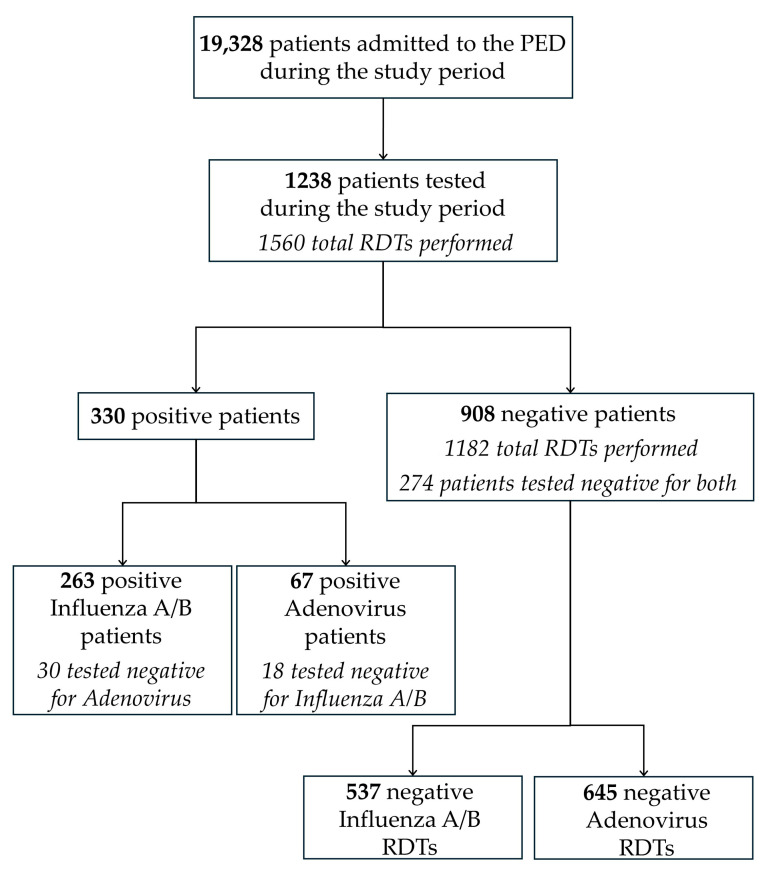
Legend: Flowchart of patient selection and test results during the study period. Of the 19,328 patients evaluated in the PED during the study period, 1238 underwent RDT testing for influenza A/B and/or adenovirus. Among them, 330 tested positive. The diagram illustrates patient inclusion and distribution according to test results. The figure also details the number of negative results for each virus. Abbreviations: PED: pediatric emergency department; RDT: rapid diagnostic test.

Patients were categorized as ‘positive’ if at least one RDT was positive, even when multiple tests were performed. Patients with negative results for all tests were included in all ‘negative’ groups. Figure 1 summarizes and illustrates the recruitment and grouping method.

The PED LOSs were compared between the two groups in terms of TTD and ATD waiting times. Additionally, the two LOS times were compared among patients with at least one positive RDT result.

### Statistical Analysis

Categorical variables are reported as absolute frequencies and percentages, while continuous variables are presented as medians and interquartile ranges. Continuous variables were compared using the Mann–Whitney U test, while categorical variables were compared using either the chi-square or Fisher’s exact test. Statistical significance was established at *p* < 0.05 for all values, which were determined using two-tailed tests. All statistical analyses were performed using GraphPad Prism version 9.1.0 for Windows (GraphPad Software, San Diego, CA, USA, www.graphpad.com) or IBM SPSS Statistics for Windows Version 21.0 (IBM Corp., Armonk, NY, USA).

## 3. Results

During the study period, 1238 patients met the inclusion criteria, accounting for 6.5% of the total 19,328 patients assessed at the IRCCS Giannina Gaslini PED. Among these patients, 1238 underwent RDTs for influenza A/B and/or adenovirus, with a total of 1560 tests performed. Of these, 330 patients (26.6%) tested positive: 263 for influenza A/B (30 of whom were negative for adenovirus) and 67 for Adenovirus (18 of whom were negative for Influenza A/B). Among 908 patients with negative results (73.4%), 274 were tested for both viruses and were negative for both.

The median age of the cohort was 3.3 years (interquartile range: 1.4–7.2 years), with a slight male predominance (58.1%). No significant differences in gender or age were observed between the two groups. No patient who was tested left the PED before being seen.

The LOS in the PED differed significantly between the two groups. Patients with at least one positive RDT had a median TTD of 217.0 min (125.0–295.5), whereas those with negative tests had a median TTD of 239.0 min (157.0–335.0). This difference was statistically significant (*p* < 0.001), and a similar difference was observed for ATD (*p* < 0.001). The main data are presented in Table 1.

These findings are further corroborated by the individual analysis of patients who underwent RDTs for influenza and adenovirus, both for TTD and ADT (Table 2 and Table 3).

Patients who tested positive for influenza had significantly shorter LOS than those patients who tested negative both for TTD and ATD (*p* = 0.006 and <0.001, respectively). Similarly, adenovirus-positive patients had shorter TTD and ATD than negative patients (*p* = 0.001 and <0.001, respectively). Additionally, no increased rate of readmission within 72 h post-discharge was observed in patients with a positive RDT across any of the populations examined.

To eliminate potential bias based on patient clinical severity, additional virus-specific analyses were performed comparing only discharged patients, who were considered clinically stable. The results obtained were consistent with those previously obtained and are reported in Supplementary Tables S1–S3.

Finally, a statistically significant difference was identified between TTD and ATD in patients with positive results, both overall and by specific etiology (Table 4).

## 4. Discussion

ED overcrowding and misuse represent intricate, multifactorial challenges that cannot be addressed solely by ED management teams alone. Effective solutions require coordinated efforts across healthcare delivery systems. This study has demonstrated an association between rapid viral testing and reduced PED LOS, suggesting its potential contribution to optimizing patient flow and, more broadly, addressing overcrowding. While the median reduction in TTD LOS was modest at the individual level, even small time savings can have a significant operational impact when applied to large patient volumes in high-throughput EDs. However, given the retrospective design of the study, these findings should be interpreted as indicating an association rather than a causal effect.

These results are consistent with previous research, which suggests that a positive RDT can expedite clinical decision-making and patient disposition in pediatric emergency settings [5,6,12,15,18,19,20]. With an analysis time of approximately 15 min and a sensitivity exceeding 85% for both viruses, as recommended by the World Health Organization for rapid tests, positive RDT results in PEDs have been associated with reduced hospital admissions, shorter LOS, and reduced hospital resource utilization [5,6,7,11,12,19,21]. Consequently, positive RDT results for adenovirus and influenza A/B in the PED setting may facilitate earlier diagnosis and disposition decisions [7,19,20]. Conversely, patients with negative RDT results likely represent cases of ongoing diagnostic uncertainty, resulting in longer observation and decision times.

However, while there is consensus on the efficacy of RDTs in pediatric populations, conflicting data persist in adult EDs [6,7,13,14,22,23,24]. Consequently, the LOS in adult EDs could not be consistently reduced, and positive RDTs for respiratory viruses should be interpreted differently when comparing adult and pediatric patients [14]. These discrepancies can be partially attributed to the fact that, while RDTs enable early diagnosis in children without further assessment, in elderly patients or those with cancer or comorbidities, a positive RDT result may indicate an increased risk of clinical deterioration, necessitating increased hospitalization and targeted therapies [6,7,9,13,23,24]. Therefore, PEDs differ substantially from adult settings in terms of disease epidemiology, patient management, and admission thresholds, which may influence the operational impact of rapid viral diagnostics. Therefore, RDTs could play a different role depending on the population in which they are used, allowing safe discharge in children and predicting clinical deterioration in adults.

Seasonal influenza often peaks, contributing significantly to overcrowding in both EDs and PEDs [8,10,13,22]. Consequently, implementing an influenza RDT may reduce the LOS in EDs and facilitate a more efficient distribution of patient flow within the department [3,6,13]. This test has been proposed for use during triage as part of a “see and treat” process, which has been widely recommended as a partial solution to ED overcrowding [1]. In adult EDs, where the efficacy of positive RDTs in reducing LOS is debated, this practice has been shown to significantly decrease LOS without increasing the bounce-back admissions rate [9,14,22]. This finding is particularly noteworthy and is also supported by the present data. In routine clinical practice, these tests were performed during the initial clinical evaluation and could be incorporated into the triage assessment workflow depending on the patient’s condition. Thus, although the ATD is logically shorter than the TTD, the difference supports the need to reorganize clinical workflows. Early test results at triage could allow physicians’ time to be redistributed without prolonging the overall LOS.

These findings are further corroborated by the lessons learned from the SARS-CoV-2 pandemic in recent years. In healthcare settings, early and rapid virus detection is crucial to implement appropriate infection control measures and promptly assign patients to suitable care pathways, particularly among vulnerable groups such as the elderly, infants, and individuals with comorbidities [14,16,25]. Together with the evidence from this study, these findings suggest that the judicious use of RDTs can facilitate more appropriate clinical and therapeutic decisions.

The observed reduction in LOS among patients with positive RDTs results in this study can be attributed to several factors. Firstly, the rapid identification of viral etiologies likely enabled more targeted management decisions, such as initiating antiviral therapy when appropriate or avoiding unnecessary antibiotic use [9,10,11,12,13,17,19,20]. Secondly, early diagnoses have allowed healthcare providers to implement timely infection control measures, thereby reducing the need for prolonged isolation and monitoring within PEDs [9,12,13]. Together, these factors contribute to more efficient patient throughput, which is essential for addressing the challenges of PED overcrowding [2,3,4]. Furthermore, conducting RDTs directly in triage could yield several positive outcomes, including cohort isolation of non-urgent, positive patients awaiting consultation, early diagnosis and discharge with increased parental satisfaction, and improved resource allocation. This would allow ED rooms and staff physicians to focus on higher-acuity patients [1,2,3,4,5,6,11,13,24].

Previous studies have documented the impact of influenza RDTs on PED patient flow, and the present results support their usefulness [5,6,12,16].

Moreover, this study is one of the first studies to analyze patients tested for adenovirus, individually. Although adenovirus is less prevalent than influenza, it can occur throughout the year without predictable seasonality, unlike influenza, and can cause severe illness in children by mimicking bacterial infections or inflammatory diseases [7,17]. While several studies have evaluated the impact of influenza rapid testing on emergency department flow, the operational implications of adenovirus rapid have not been well investigated, particularly in pediatric emergency settings. Consequently, it is crucial to consider all potential viral etiologies to enhance the accuracy of etiological diagnoses and expedite clinical therapeutic decision-making. However, the relatively small number of adenovirus-positive patients requires the results of subgroup analyses to be interpreted with caution.

Notably, this analysis showed that the rate of return visits within 72 h was comparable between patients with positive and negative rapid antigen test results. This finding suggests that using RDTs does not compromise patient safety or result in premature discharge. Previous studies have reported similar outcomes, indicating that RDTs do not increase the likelihood of adverse events or return visits [6]. Furthermore, it has been observed that the prevalence of bacterial infection is lower in young children aged three to 36 months who test positive for viral infection [5].

While the present study provides evidence supporting the use of RDTs in reducing PED LOS, it is important to consider the broader implications of PED overcrowding. Overcrowding is a complex issue influenced by factors such as patient inflow, hospital capacity, and systemic healthcare dynamics [2,3,4]. Therefore, RDTs should be considered as a part of broader system-level strategies aimed at improving patient flow and reducing emergency department overcrowding.

### Limitations

Firstly, as this was a retrospective health services study, data regarding clinical confounders and variables such as comorbidities, disease severity, and the rationale behind clinicians’ decisions to perform RDTs were not systematically recorded. As the analyses were exploratory and focused on predefined comparisons between positive and negative RDT groups, no correction for multiple testing was applied. Furthermore, multivariable adjustment for potential confounders could not be performed as several clinical variables were not available. Therefore, secondary comparisons should be interpreted with caution. However, the aim was to capture the overall effect of rapid testing within routine clinical practice.

Secondly, PED crowding, the triage codes, and the waiting times for patients awaiting admission were not considered, since in this context, the LOS in the PED is influenced not only by diagnostic and decision-making efficiency but also by departmental capacity to accept patients.

Thirdly, while the median reduction observed at the individual level was modest, such time savings could be operationally significant in high-volume emergency settings. Therefore, future prospective studies are required to determine whether this reduction translates into meaningful improvements. Furthermore, only two of the most prevalent viruses were included. Many other viruses, such as parainfluenza, bocavirus, and metapneumovirus, were not tested because, to date, they do not yet have dedicated RDTs.

Fourthly, as this study was conducted in a single tertiary pediatric emergency department, the findings should not be generalized to other healthcare systems or settings with different patient populations, testing policies, or workflow organization.

Lastly, the study lacked data on viral-bacterial coinfection.

## 5. Conclusions

These findings suggest an association between positive RDT results and shorter PED LOS without an associated increase in the risk of return visits. While this may offer potential support for patient flow management, conclusions regarding the overall effectiveness of RDTs in reducing overcrowding should be interpreted cautiously.

The early diagnosis of the cause of illness in febrile pediatric patients presenting with respiratory symptoms using RDTs could be a partial solution to support patient flow management and operational efficiency in PEDs. Further clinical trials, including triage codes, PED crowding indices, bed availability, and the implementation of RDTs for additional viruses, are necessary to evaluate the role of rapid diagnostic technologies in managing patient flow within PEDs.

## Figures and Tables

**Table 1:** Demographic and clinical characteristics of tested patients.

	Total Patients *n* = 1238	Positive Patients *n* = 330	Negative Patients *n* = 908	*p*
Age in years, median (IQR)	3.3 (1.4–7.2)	3.6 (1.8–6.8)	3.2 (1.3–7.5)	0.20
Sex, male (%)	722 (58.1)	192 (58.2)	530 (58.4)	0.95
TTD in minutes, median (IQR)	232.5 (147.0–324.0)	217.0 (125.0–295.5)	239.0 (157.0–335.0)	<0.001
ATD in minutes, median (IQR)	132.0 (60.0–215.0)	66.0 (37.0–154.5)	148.5 (84.0–227.0)	<0.001
Readmission, yes (%)	58 (4.7)	14 (4.2)	44 (4.8)	0.65

Continuous variables were compared using the Mann–Whitney U-test; categorical variables were compared using the chi-square or Fisher’s exact test. Abbreviations: TTD: triage-to-discharge time; ATD: assignment-to-discharge time; IQR: interquartile range.

**Table 2:** Clinical characteristics of patients tested for Influenza A/B.

	Total Influenza A/B Patients *n* = 908	Positive Patients *n* = 263	Negative Patients *n* = 645	*p*
Age in years, median (IQR)	3.6 (1.4–7.7)	4.0 (2.0–7.6)	3.2 (1.7–7.9)	0.18
Sex, male (%)	527 (58.0)	148 (56.2)	379 (58.7)	0.49
TTD in minutes, median (IQR)	229.5 (144.0–323.0)	220.0 (125.0–297.0)	235.0 (149.0–334.0)	0.006
ATD in minutes, median (IQR)	126.0 (56.0–206.0)	64.0 (36.0–150.0)	144.0 (82.0–225.5)	<0.001
Readmission, yes (%)	39 (4.3)	13 (4.9)	26 (4.0)	0.53

Continuous variables were compared using the Mann–Whitney U-test; categorical variables were compared using the chi-square or Fisher’s exact test. Abbreviations: TTD: triage-to-discharge time; ATD: assignment-to-discharge time; IQR: interquartile range.

**Table 3:** Clinical characteristics of patients tested for Adenovirus.

	Total Adenovirus Patients *n* = 604	Positive Patients *n* = 67	Negative Patients *n* = 537	*p*
Age in years, median (IQR)	2.8 (1.2–6.3)	2.2 (1.3–4.9)	2.9 (1.2–6.9)	0.09
Sex, male (%)	353 (58.4)	44 (65.1)	309 (57.5)	0.20
TTD in minutes, median (IQR)	251.5 (169.0–346.5)	215.0 (132.5–281.0)	257.0 (178.0–352.0)	0.001
ATD in minutes, median (IQR)	154.5 (88.0–242.0)	77.0 (45.5–175.0)	163.0 (100.0–249.0)	<0.001
Readmission, yes (%)	28 (4.6)	1 (1.5)	27 (5.0)	0.19

Continuous variables were compared using the Mann–Whitney U-test; categorical variables were compared using the chi-square or Fisher’s exact test. Abbreviations: TTD: triage-to-discharge time; ATD: assignment-to-discharge time; IQR: interquartile range.

**Table 4:** Comparison of TTD and ATD times among positive patients.

	TTD in Minutes, Median (IQR)	ATD in Minutes, Median (IQR)	*p*
Total positive patients *n* = 330	217.0 (125.0–295.5)	66.0 (37.0–154.5)	<0.001
Flu A/B-positive patients *n* = 263	220.0 (125.0–297.0)	64.0 (36.0–150.0)	<0.001
Adenovirus-positive patients *n* = 67	215.0 (132.5–281.0)	77.0 (45.5–175.0)	<0.001

Comparison between TTD and ADT in positive patients. Abbreviations: TTD: triage-to-discharge time; ATD: assignment-to-discharge time; IQR: interquartile range.

## Data Availability

The datasets used and analyzed in this paper are available from the corresponding author on reasonable request.

## Supplementary Materials

The following supporting information can be downloaded at https://www.mdpi.com/article/10.3390/healthcare14070925/s1. Table S1. Demographic and clinical characteristics of dismissed tested patients. Table S2. Clinical characteristics of dismissed patients tested for Influenza A/B. Table S3. Clinical characteristics of dismissed patients tested for Adenovirus.

## Abbreviations

The following abbreviations are used in this manuscript:

ATD	Assignment-to-discharge time
ED	Emergency Department
IRCCS	Istituto di Ricerca e Cura a Carattere Scientifico
LOS	Length of Stay
PED	Pediatric Emergency Department
RDT	Rapid Diagnostic Test
RT-PCR	Real-Time Polymerase Chain Reaction
SARS-CoV-2	Severe Acute Respiratory Syndrome Corona Virus 2
TAT	Turnaround Time
TTD	Triage-to-discharge time
