# Supplementary material for: Impact of Rapid Viral Testing on Patient Flow and Length of Stay in a Tertiary Pediatric Emergency Department
Source: Healthcare (Basel). 2026 Apr 2;14(7):925. doi: 10.3390/healthcare14070925 (PMC13073589; doi:10.3390/healthcare14070925)
Supplement: Supplementary file 1 [file healthcare-14-00925-s001.zip › healthcare-4177626-supplementary.pdf]

Table S1. Demographic and clinical characteristics of dismissed tested patients.

	**Total Patients** n = 839	**Positive Patients** n = 273	**Negative Patients** n = 565	*p*
Age in years, *median (IQR)*	3.5 (1.5-7.5)	3.7 (1.8-7.0)	3.3 (1.3-7.9)	0.43
Sex, *male (%)*	476 (56.7)	157 (57.5)	319 (56.4)	0.77
TTD in minutes, *median (IQR)*	224.0 (141.0-307.5)	198.0 (124.0-288.0)	231.0 (149.0-321.0)	**0.001**
ATD in minutes, *median (IQR)*	106.5 (50.0-177.5)	54.0 (36.0-136.0)	124.0 (69.0-198.0)	**< 0.001**

Abbreviations: *TTD: triage-to-discharge time; ATD: admission-to-discharge time; IQR: interquartile range; RDT: rapid diagnostic test.*

Table S2. Clinical characteristics of dismissed patients tested for Influenza A/B.

	**Total Influenza A/B Patients** n = 633	**Positive Patients** n = 219	**Negative Patients** n = 414	*p*
Age in years, *median (IQR)*	3.7 (1.6-8.1)	4.2 (2.1-7.7)	3.4 (1.3-8.1)	0.08
Sex, *male (%)*	355 (56.0)	121 (55.2)	234 (56.5)	0.75
TTD in minutes, *median (IQR)*	220.0 (134.0-305.0)	202.0 (123.5-288.5)	223.5 (145.0-318.5)	**0.017**
ATD in minutes, *median (IQR)*	102.0 (48.0-172.0)	54.0 (34.0-133.0)	122.5 (64.0-192.5)	**< 0.001**

Abbreviations: *TTD: triage-to-discharge time; ATD: admission-to-discharge time; IQR: interquartile range; RDT: rapid diagnostic test.*

Table S3. Clinical characteristics of dismissed patients tested for Adenovirus.

	**Total Adenovirus Patients** n = 364	**Positive Patients** n = 54	**Negative Patients** n = 310	*p*
Age in years, *median (IQR)*	2.8 (1.3-6.3)	2.1 (1.3-4.9)	3.1 (1.3-7.3)	0.07
Sex, *male (%)*	206 (56.5)	36 (66.6)	170 (54.8)	0.10
TTD in minutes, *median (IQR)*	247.0 (166.5-335.5)	178.0 (128.0-280.5)	252.5 (175.0-338.0)	**0.003**
ATD in minutes, *median (IQR)*	130.0 (73.0-205.0)	57.5 (44.0-164.0)	133.0 (85.0-218.0)	**< 0.001**

Abbreviations: *TTD: triage-to-discharge time; ATD: admission-to-discharge time; IQR: interquartile range; RDT: rapid diagnostic test.*
